# EWAS Open Platform: integrated data, knowledge and toolkit for epigenome-wide association study

**DOI:** 10.1093/nar/gkab972

**Published:** 2021-10-30

**Authors:** Zhuang Xiong, Fei Yang, Mengwei Li, Yingke Ma, Wei Zhao, Guoliang Wang, Zhaohua Li, Xinchang Zheng, Dong Zou, Wenting Zong, Hongen Kang, Yaokai Jia, Rujiao Li, Zhang Zhang, Yiming Bao

**Affiliations:** National Genomics Data Center, Beijing Institute of Genomics, Chinese Academy of Sciences / China National Center for Bioinformation, Beijing 100101, China; CAS Key Laboratory of Genome Sciences and Information, Beijing Institute of Genomics, Chinese Academy of Sciences, Beijing 100101, China; University of Chinese Academy of Sciences, Beijing 100049, China; National Genomics Data Center, Beijing Institute of Genomics, Chinese Academy of Sciences / China National Center for Bioinformation, Beijing 100101, China; CAS Key Laboratory of Genome Sciences and Information, Beijing Institute of Genomics, Chinese Academy of Sciences, Beijing 100101, China; University of Chinese Academy of Sciences, Beijing 100049, China; National Genomics Data Center, Beijing Institute of Genomics, Chinese Academy of Sciences / China National Center for Bioinformation, Beijing 100101, China; CAS Key Laboratory of Genome Sciences and Information, Beijing Institute of Genomics, Chinese Academy of Sciences, Beijing 100101, China; National Genomics Data Center, Beijing Institute of Genomics, Chinese Academy of Sciences / China National Center for Bioinformation, Beijing 100101, China; CAS Key Laboratory of Genome Sciences and Information, Beijing Institute of Genomics, Chinese Academy of Sciences, Beijing 100101, China; National Genomics Data Center, Beijing Institute of Genomics, Chinese Academy of Sciences / China National Center for Bioinformation, Beijing 100101, China; CAS Key Laboratory of Genome Sciences and Information, Beijing Institute of Genomics, Chinese Academy of Sciences, Beijing 100101, China; University of Chinese Academy of Sciences, Beijing 100049, China; National Genomics Data Center, Beijing Institute of Genomics, Chinese Academy of Sciences / China National Center for Bioinformation, Beijing 100101, China; CAS Key Laboratory of Genome Sciences and Information, Beijing Institute of Genomics, Chinese Academy of Sciences, Beijing 100101, China; University of Chinese Academy of Sciences, Beijing 100049, China; National Genomics Data Center, Beijing Institute of Genomics, Chinese Academy of Sciences / China National Center for Bioinformation, Beijing 100101, China; CAS Key Laboratory of Genome Sciences and Information, Beijing Institute of Genomics, Chinese Academy of Sciences, Beijing 100101, China; University of Chinese Academy of Sciences, Beijing 100049, China; National Genomics Data Center, Beijing Institute of Genomics, Chinese Academy of Sciences / China National Center for Bioinformation, Beijing 100101, China; CAS Key Laboratory of Genome Sciences and Information, Beijing Institute of Genomics, Chinese Academy of Sciences, Beijing 100101, China; National Genomics Data Center, Beijing Institute of Genomics, Chinese Academy of Sciences / China National Center for Bioinformation, Beijing 100101, China; CAS Key Laboratory of Genome Sciences and Information, Beijing Institute of Genomics, Chinese Academy of Sciences, Beijing 100101, China; National Genomics Data Center, Beijing Institute of Genomics, Chinese Academy of Sciences / China National Center for Bioinformation, Beijing 100101, China; CAS Key Laboratory of Genome Sciences and Information, Beijing Institute of Genomics, Chinese Academy of Sciences, Beijing 100101, China; University of Chinese Academy of Sciences, Beijing 100049, China; National Genomics Data Center, Beijing Institute of Genomics, Chinese Academy of Sciences / China National Center for Bioinformation, Beijing 100101, China; CAS Key Laboratory of Genome Sciences and Information, Beijing Institute of Genomics, Chinese Academy of Sciences, Beijing 100101, China; University of Chinese Academy of Sciences, Beijing 100049, China; National Genomics Data Center, Beijing Institute of Genomics, Chinese Academy of Sciences / China National Center for Bioinformation, Beijing 100101, China; CAS Key Laboratory of Genome Sciences and Information, Beijing Institute of Genomics, Chinese Academy of Sciences, Beijing 100101, China; National Genomics Data Center, Beijing Institute of Genomics, Chinese Academy of Sciences / China National Center for Bioinformation, Beijing 100101, China; CAS Key Laboratory of Genome Sciences and Information, Beijing Institute of Genomics, Chinese Academy of Sciences, Beijing 100101, China; National Genomics Data Center, Beijing Institute of Genomics, Chinese Academy of Sciences / China National Center for Bioinformation, Beijing 100101, China; CAS Key Laboratory of Genome Sciences and Information, Beijing Institute of Genomics, Chinese Academy of Sciences, Beijing 100101, China; University of Chinese Academy of Sciences, Beijing 100049, China; National Genomics Data Center, Beijing Institute of Genomics, Chinese Academy of Sciences / China National Center for Bioinformation, Beijing 100101, China; CAS Key Laboratory of Genome Sciences and Information, Beijing Institute of Genomics, Chinese Academy of Sciences, Beijing 100101, China; University of Chinese Academy of Sciences, Beijing 100049, China

## Abstract

Epigenome-Wide Association Study (EWAS) has become a standard strategy to discover DNA methylation variation of different phenotypes. Since 2018, we have developed EWAS Atlas and EWAS Data Hub to integrate a growing volume of EWAS knowledge and data, respectively. Here, we present EWAS Open Platform (https://ngdc.cncb.ac.cn/ewas) that includes EWAS Atlas, EWAS Data Hub and the newly developed EWAS Toolkit. In the current implementation, EWAS Open Platform integrates 617 018 high-quality EWAS associations from 910 publications, covering 51 phenotypes, 275 diseases and 104 environmental factors. It also provides well-normalized DNA methylation array data and the corresponding metadata from 115 852 samples, which involve 707 tissues, 218 cell lines and 528 diseases. Taking advantage of integrated knowledge and data in EWAS Atlas and EWAS Data Hub, EWAS Open Platform equips with EWAS Toolkit, a powerful one-stop site for EWAS enrichment, annotation, and knowledge network construction and visualization. Collectively, EWAS Open Platform provides open access to EWAS knowledge, data and toolkit and thus bears great utility for a broader range of relevant research.

## INTRODUCTION

With the explosive growth of epigenome-wide association studies (EWAS), huge amounts of data and knowledge related to EWAS have been accumulated (1). Since these data hold great potential for clinical translations, a standardized platform for data archive, retrieval and exploration is indispensable. In order to identify potential biomarkers for human healthcare and disease treatment (2–6), a large number of EWAS associations have been reported in publications, posing great challenges in literature curation and knowledge synthesis. To this end, valuable efforts have been made worldwide, with the purpose to develop several databases and tools in aid of EWAS data integration and analysis (7–12). In 2018, we, the EWAS team of the National Genomics Data Center (NGDC) (13,14), launched EWAS Atlas (https://ngdc.cncb.ac.cn/ewas/atlas), a EWAS knowledgebase hosting manually curated high-quality EWAS associations (15). Subsequently, we further constructed EWAS Data Hub (https://ngdc.cncb.ac.cn/ewas/datahub), a data portal for collecting and normalizing DNA methylation array data as well as archiving associated metadata (16).

**Figure 1.**
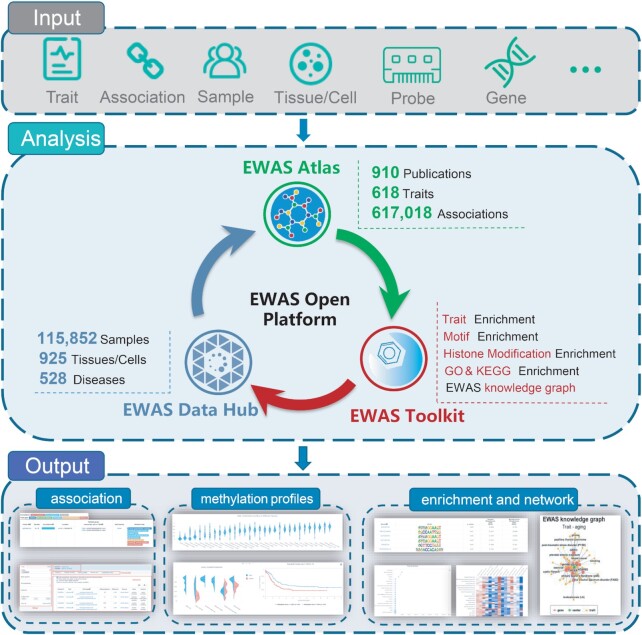
Schematic overview of EWAS Open Platform data processing workflow.

Since the first release of EWAS Atlas, there are over 28 000 visitors with a total of 127 000 accesses, along with >100 emails and phone calls from worldwide users (Table 1). To promote the data-to-bedside research to inform diagnosis and guide treatments by, for example, the identification of more indicative biomarkers from data (16), we developed EWAS Toolkit (https://ngdc.cncb.ac.cn/ewas/toolkit), a web-based tool suite for EWAS downstream analyses that comprise a series of online services for EWAS enrichment & annotation and network visualization, on the basis of integrated knowledge and data from EWAS Atlas, EWAS Data Hub, and the Roadmap Epigenomics Project (17). Pulling EWAS Atlas and EWAS Data Hub that have significant updates in the past several years, together with the recently developed EWAS Toolkit, here we introduce EWAS Open Platform (https://ngdc.cncb.ac.cn/ewas/) (Figure 1), serving as a one-stop site to deliver a portfolio of services for EWAS data, knowledge and toolkit.

**Table 1.** Main updates of EWAS open platform

	EWAS Atlas (2019)/EWAS Data Hub (2020)	EWAS Open Platform (2021) (EWAS Atlas/ EWAS Data Hub)
**Data and Information**		
Associations	329 172/NA	617 018/NA
Traits	305/NA	618/NA
Cohorts	1830/NA	3382/NA
Tissues or Cells	112/470	193/925
Studies	898/NA	1437/NA
Publications	649/NA	910/NA
Samples	NA/75 344	NA/115 852
Disease	128/306	275/528
Fields	NA/178	NA/242
**Toolkit**		
Enrichments	Trait, Genomic location, GO, KEGG and Motif enrichment	
Annotations	Chromatin state, Histone modification, Tissue methylation and Expression regulation	
Network	Knowledge graph	
**Usage**		
Visitors^a^	28 879	
Visits^a^	127 847	
Citations^b^	84	

^a^Data as of 12 September 2021.

^b^Data retrieved from Google Scholar, as of 12 September 2021.

## MAJOR COMPONENTS AND UPDATES

EWAS Open Platform is an open platform for epigenome-wide association studies that incorporates three components: EWAS Data Hub for data collection and standardized normalization, EWAS Atlas for knowledge extraction and curation, and EWAS Toolkit for downstream analysis and visualization. Each component is a stand-alone database or web server.

### EWAS Atlas

As a curated knowledgebase of EWAS Open Platform, EWAS Atlas has been enriched by adding a total of 287 864 EWAS associations manually curated from 509 publications, nearly doubling the number of associations by comparison with the first release in 2018. As a result, EWAS Atlas currently houses a total of 617 018 high-quality EWAS associations reported in 910 publications, involving 618 traits, 1437 studies, 3382 cohorts and 193 tissues/cell types (Table 1). Users can browse the associations in light of trait, probe, gene, study and publication, which are displayed in five panels on the browse page. In addition to efficient search engine and handy download services, EWAS Atlas provides expanded panels to show detailed and quantitative information, such as the rank, *P* value, effect size, trait and methylation value.

### EWAS Data Hub

As a normalized data portal of EWAS Open Platform, EWAS Data Hub features comprehensive integration of all available datasets generated by Infinium HumanMethylation450 and MethylationEPIC BeadChip from GEO (18), TCGA (19), ArrayExpress (20) and ENCODE (21). In addition, it adopts a set of curation processes to eliminate batch effects and improve data quality (22). EWAS Data Hub has been significantly updated by including 40 508 high-quality samples of DNA methylation array data and metadata since the first release in 2019. Currently, a total of 115 852 samples are stored in EWAS Data Hub, covering 242 fields, 925 tissue/cells (including 218 cell lines) and 528 diseases (Table 1).

### EWAS Toolkit

**Figure 2.**
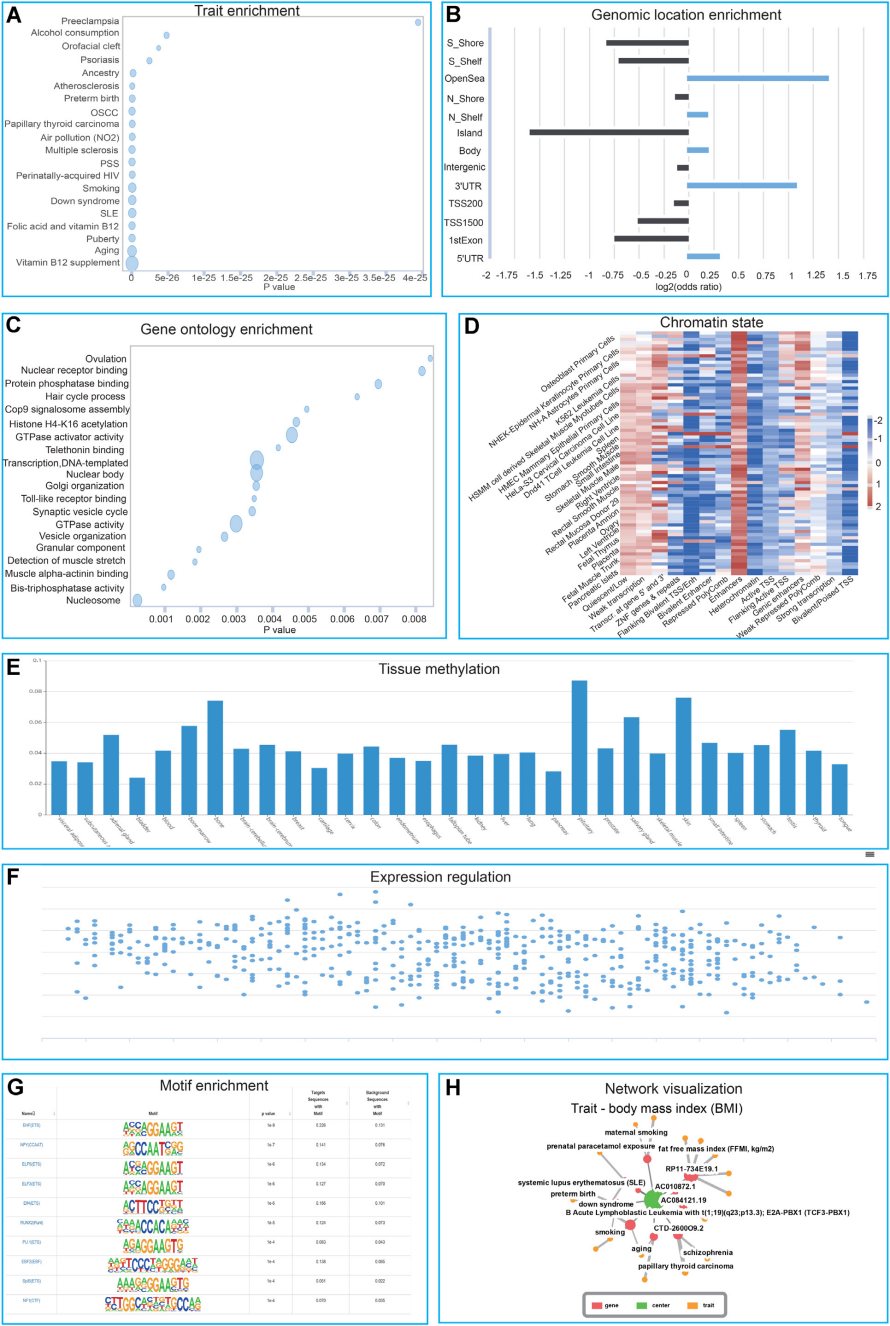
Analysis panels of EWAS Toolkit. (**A**) Trait enrichment, showing that specific traits are significantly enriched. (**B**) Genomic location enrichment, demonstrating that DNA methylation sites related to vitamin B12 supplementation are enriched in Non-CpG island and 3'UTR regions. (**C**) Gene ontology enrichment, showing the enriched GO terms of genes near the input probe set. (**D**) Chromatin state, showing the significant enrichment in the enhancer region. (**E**) Tissue methylation, providing methylation levels across tissues of the input probe site. (**F**) Expression regulation, showing the relationship between the methylation level of the input site and the expression level of nearby genes. (**G**) Motif enrichment, displaying the enriched motifs and their associated transcription factors in the vicinity of the input probe set. (**H**) Network visualization, by taking BMI as an example.

As an indispensable component of EWAS Open Platform, EWAS Toolkit is a new powerful one-stop analysis service for EWAS downstream analysis. Currently, EWAS Toolkit firstly features trait enrichment and network visualization by leveraging 617 018 high-quality associations from 910 publications in EWAS Atlas. It has been widely used in the retrieval and discovery of epigenetics biomarkers since its release (23–25). At the same time, combined with the methylation and expression profile data in the EWAS Data Hub, we provide tissue methylation and expression regulation annotations across 31 tissues. In addition, EWAS Toolkit integrates knowledge and data, organically combines EWAS Atlas and EWAS Data Hub, and provides users with a wide range of analysis and visualization including enrichment, annotation and network visualization (Figure 2).

### Enrichment and annotation

EWAS Toolkit allows users to provide probes and trait term as input. Analyzed results are categorized into five enrichment sections (trait, genomic location, GO, KEGG and motif enrichment) and four annotation sections (chromatin state, histone modification, methylation and expression regulation annotation). DNA methylation probes related to vitamin B12 supplementation reported in the literature are provided as an example in the web page of EWAS Toolkit (26). To facilitate users for further analysis, all results can be packaged and downloaded.

#### Enrichment

Trait enrichment analysis is based on the curated associations related to traits in the EWAS Atlas. Weighted Fisher's exact test was used to compute the probability of co-occurrence between input DNA methylation probes and trait-related DNA methylation probes (Figure 2A). For genomic location enrichment, we pre-defined 13 location categories based on the location relative to gene and CpG island. The result of genomic location enrichment shows the enrichment of input sites in different regions of the genome (Figure 2B). Because the number of probes designed for each gene on the DNA methylation array is not equal, the use of traditional Fisher's exact test or chi-square test can lead to bias in GO and KEGG enrichment results (27,28). To address this issue, we adopted the algorithm designed by Phipson *et al.* based on the Wallenius non-central hypergeometric distribution (29), which can calculate and add the number of probes designed on each gene as prior knowledge to the inspection process. The ‘gometh’ function from the R package ‘missMethyl’ was used in this implementation (29). The GO and KEGG enrichment results show the enrichment of genes near the input probes in the GO entry and KEGG pathway, respectively (Figure 2C). Motif refers to a characteristic sequence with biological significance, such as binding sequence of regulatory factors like transcription factors. Motif enrichment near DNA methylation can aid the identification of proteins that interact with DNA methylation. The HOMER (Hypergeometric Optimization of Motif EnRichment, http://homer.ucsd.edu/homer) knowledgebase, which leverages a massive amount of chromatin immunoprecipitation data for transcription factor motif identification (30), was used for motif enrichment in EWAS Toolkit (Figure 2G).

#### Annotation

The state of histone modification, a chemical modification that occurs on histones, can influence the structure of chromatin, thereby affecting gene expression. For histone modification enrichment, we used the histone modification data of 127 cells and tissues in the Roadmap Epigenomics Project (17). Chromatin state refers to the regulatory function and current activity of a region of the genome. It is usually predicted by histone modification, DNA methylation, and gene expression. For the enrichment of chromatin states, we used the chromatin state data by the Roadmap Epigenomics Project to identify a total of 15 chromatin states (Figure 2D). EWAS Toolkit provides tissue methylation and expression regulation annotations based on EWAS Data Hub. The annotation of tissue methylation displays the input probe's tissue-specificity (tau) (16) as well as the DNA methylation level in 31 tissues (Figure 2E). The relationship between methylation level of the input probes and expression level of nearby genes is depicted as expression regulation (Figure 2F).

### Network visualization

In order to assist users to explore the hierarchical associations between traits and genes in a visualized network, EWAS Toolkit is capable to provide an EWAS knowledge graph by linking any given trait/gene to its associated genes/traits. Users can select one or two ways to calculate the correlation coefficient between genes and traits. The first is based on the number of publications reporting on the relations between a gene and a trait. The second is based on the inferred associations from data, taking the sum of the number of associations between all probes on the gene and traits as the association coefficient. On the knowledge graph page (https://ngdc.cncb.ac.cn/ewas/network), users can specify various parameters, such as the central node, the number of layers displayed, the maximum number of edges from each node, and the calculation method of the correlation coefficient. The network structure can be updated in real time when any parameter is changed (Figure 2H).

## DISCUSSION AND FUTURE DEVELOPMENTS

In this study, we present EWAS Open Platform, an integrated open platform for EWAS data storage and download, knowledge collection and browsing, and downstream analysis and visualization. Apart from adding 287 864 EWAS associations from 509 publications to EWAS Atlas and 40 508 high-quality samples to EWAS Data Hub, EWAS Open Platform now contains a data analysis component, EWAS Toolkit, that supports various online analyses for EWAS enrichment, annotation, and network visualization. In particular, EWAS Toolkit has generated the first re-mining analysis platform based on enormous amounts of knowledge data from literatures, integrating trait enrichment and EWAS network visualization paired with knowledge graph. Future developments of EWAS Open Platform are frequent updates of these three components and enhancement of data sharing and information flow between EWAS Data Hub, EWAS Atlas and EWAS toolkit. For EWAS Atlas, we will optimize the curation model, accept community-curated annotations combined with expert review, and improve curation efficiency and quality. For EWAS data Hub, we will update the reference DNA methylation profile and improve cell line browsing and display based on the newly added cell line data. For EWAS Toolkit, we will use graph theory and machine learning methods to facilitate in-depth mining of knowledge graph, as well as analyze and predict complex relationships between phenotypes, environments, and behaviors. In addition, by combining methylation databases in NGDC, particularly MethBank (31) and scMethBank (32), EWAS Open Platform will provide easy and open access to more comprehensive data, knowledge and toolkit.

## DATA AVAILABILITY

EWAS Open Platform is an integrated database and analysis platform for Epigenome-Wide Association Study which is freely available online and all data can be accessed at https://ngdc.cncb.ac.cn/ewas.
